# The South African Tuberculosis Care Cascade: Estimated Losses and Methodological Challenges

**DOI:** 10.1093/infdis/jix335

**Published:** 2017-11-06

**Authors:** Pren Naidoo, Grant Theron, Molebogeng X Rangaka, Violet N Chihota, Louise Vaughan, Zameer O Brey, Yogan Pillay

**Affiliations:** 1 Desmond Tutu TB Centre, Department of Paediatrics and Child Health, Faculty of Medicine and Health Sciences, Stellenbosch University, Cape Town, South Africa;; 2 DST/NRF Centre of Excellence for Biomedical Tuberculosis Research and; 3 MRC Centre for Tuberculosis Research, Division of Molecular Biology and Human Genetics, Faculty of Medicine and Health Sciences, Stellenbosch University, Cape Town, South Africa;; 4 Wellcome Centre for Infectious Disease Research in Africa, Institute of Infectious Diseases and Molecular Medicine, University of Cape Town, Cape Town,South Africa;; 5 Implementation Research Division, Aurum Institute, Johannesburg, South Africa;; 6 School of Public Health, Faculty of Health Sciences, University of the Witwatersrand, Johannesburg, South Africa;; 7 HIV/AIDS, TB, and Maternal and Child Health Branch, National Department of Health, Pretoria, South Africa; 8 Bill and Melinda Gates Foundation, Seattle, Washington;; 9 Institute of Global Health, University College London, London, United Kingdom;

**Keywords:** Tuberculosis, care cascade, continuum of care, case-finding, initial loss to follow-up, treatment success

## Abstract

**Background:**

While tuberculosis incidence and mortality are declining in South Africa, meeting the goals of the End TB Strategy requires an invigorated programmatic response informed by accurate data. Enumerating the losses at each step in the care cascade enables appropriate targeting of interventions and resources.

**Methods:**

We estimated the tuberculosis burden; the number and proportion of individuals with tuberculosis who accessed tests, had tuberculosis diagnosed, initiated treatment, and successfully completed treatment for all tuberculosis cases, for those with drug-susceptible tuberculosis (including human immunodeficiency virus (HIV)–coinfected cases) and rifampicin-resistant tuberculosis. Estimates were derived from national electronic tuberculosis register data, laboratory data, and published studies.

**Results:**

The overall tuberculosis burden was estimated to be 532005 cases (range, 333760–764480 cases), with successful completion of treatment in 53% of cases. Losses occurred at multiple steps: 5% at test access, 13% at diagnosis, 12% at treatment initiation, and 17% at successful treatment completion. Overall losses were similar among all drug-susceptible cases and those with HIV coinfection (54% and 52%, respectively, successfully completed treatment). Losses were substantially higher among rifampicin- resistant cases, with only 22% successfully completing treatment.

**Conclusion:**

Although the vast majority of individuals with tuberculosis engaged the public health system, just over half were successfully treated. Urgent efforts are required to improve implementation of existing policies and protocols to close gaps in tuberculosis diagnosis, treatment initiation, and successful treatment completion.

South Africa is one of 6 countries accounting for 60% of the global tuberculosis burden [1]. In 2015 there were an estimated 454000 incident cases, at a rate of 834 cases per 100000 population. It has the highest burden of human immunodeficiency virus (HIV) co-infected cases globally, estimated at 258000 [1]. Multidrug-resistant tuberculosis (MDR-TB) prevalence rates have remained stable, at 2.9%, in the 2001–2002 survey, compared with 2.8% in 2012–2014, but the rate of rifampicin resistance (RIF-R) has increased, from 3.4% to 4.6% [2]. While estimated tuberculosis incidence rates and mortality appear to be decreasing (Figure 1), the current rate of decline is too slow to meet the 2030 Sustainable Development Goals or 2035 End TB Strategy targets [3]. Extrapolating from World Health Organization estimates [4], by 2030 and 2035, tuberculosis incidence rates for South Africa would need to decrease to 167 and 83 cases per 100000 population, respectively, and mortality would need to decrease to 9800 and 4900 cases, respectively.

**Figure 1.**
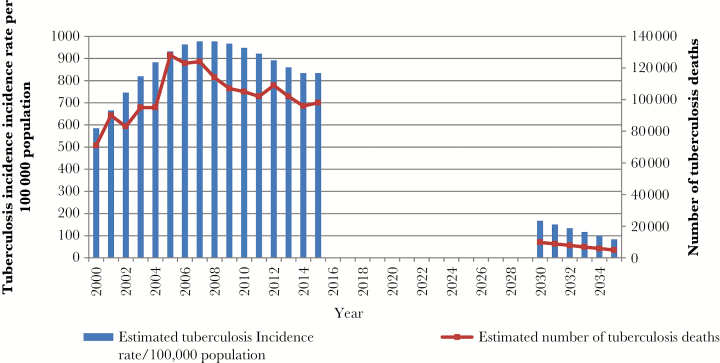
Estimated tuberculosis incidence and mortality in South Africa and projected sustainable development goals and End TB Strategy targets. Data on the estimated tuberculosis incidence rates and mortality for 2000 to 2015 are from the revised time series analysis of global tuberculosis burden published by the World Health Organization in 2016 [4]. Projected figures for 2030–2035 are based on targets relative to the 2015 estimates and assume a straight-line decline in tuberculosis incidence rates and mortality in this period.

Reaching these ambitious targets demands a more invigorated response from the National TB Programme in South Africa. The historical focus of the National TB Programme has been on treatment success rates, which fail to reflect upstream losses contributed by individuals who do not access health services and by those for whom tuberculosis is not diagnosed, notified, and treated. A better understanding of the continuum of care (also known as the “care cascade”) can provide the evidence needed on where to target interventions to reduce attrition along the pathway to successful treatment. This approach has previously been used in South Africa to assess and address implementation challenges in the program to prevent mother-to-child HIV transmission [5–7] and in antiretroviral treatment services [8].

We undertook an analysis of the tuberculosis care cascade in South Africa to assess the number and proportion of individuals with tuberculosis who accessed tests, were diagnosed with tuberculosis, initiated treatment, and successfully completed treatment, to quantify losses at each step for (1) all tuberculosis cases, (2) drug-susceptible (DS) tuberculosis cases, (3) HIV-coinfected DS tuberculosis cases, and (4) RIF-R tuberculosis cases.

## MethodS

### Setting

South Africa has a district health system, with 52 districts among its 9 provinces. There are 3500 public primary healthcare facilities [9], equivalent to 1 facility per 15000 population. Only 18.4% of the population was covered by private medical aid schemes in 2013 [10], with the balance largely dependent on the public health sector.

The majority of tuberculosis tests (93%) [11] and almost all treatment are provided within the public health sector in South Africa. Individuals treated in the mining sector and correctional services are reported to the South African National Department of Health. Tuberculosis diagnostic and treatment services are provided free of user costs in the public sector.

The National Health Laboratory Services, a parastatal organization, provides tuberculosis diagnostic services, mostly through centralized laboratories. Historically, sputum microscopy formed the basis of tuberculosis diagnosis, with liquid culture (Bactec MGIT 960) available for smear-negative, HIV-coinfected new cases and culture and drug susceptibility testing (conventional or GenoType MTBDRplus, Hain Lifescience, Nehren, Germany) available for previously treated cases. Xpert MTB/RIF (hereafter, “Xpert”; Cepheid, Sunnyvale, CA) was phased in during 2011, replacing smear microscopy for all presumptive tuberculosis cases.

Standardized first-line tuberculosis treatment is provided at a primary care level. Standardized second-line tuberculosis treatment is initiated mostly in tuberculosis hospitals, with decentralization to the primary healthcare level in some areas. All individuals receiving treatment have standard clinical records completed; data are summarized in paper-based registers at the facility level and captured into the electronic tuberculosis register (ETR) or the electronic drug-resistant tuberculosis register at the subdistrict levels. Subdistrict reports are dispatched to the district level and then to provincial and national levels.

### Estimation Method and Data Sources

We estimated the number of cases at each step of the care cascade for the following: (1) all tuberculosis cases, (2) DS tuberculosis cases, (3) HIV-coinfected DS tuberculosis cases, and (4) RIF-R cases. We elected to do the analysis for the 2013 cohorts because this was the most recent year for which a cleaned, deduplicated national data set for DS tuberculosis cases was available from the ETR and for which RIF-R tuberculosis treatment outcomes were available from the electronic drug-resistant tuberculosis register.

**Figure 2.**
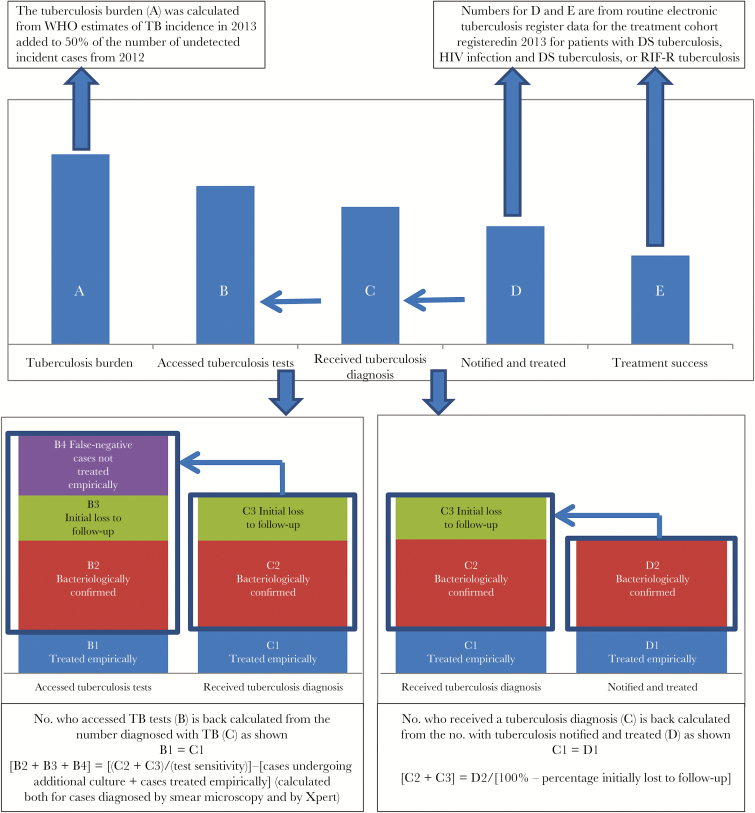
Approach to estimating the number of cases at each step in the tuberculosis care cascade. Abbreviations: DS, drug susceptible; HIV, human immunodeficiency virus; RIF-R, rifampicin resistant; WHO, World Health Organization.

In addition to the data sources described above, we used summary district data from the National Health Laboratory Services on tuberculosis testing and published literature to estimate the numbers of cases at each step or the gaps between successive steps. Figure 2 summarizes the conceptual approach, and Table 1 describes the detailed methods and calculations used to estimate each of the steps described below.

**Table 1.** Estimation of the Number of Cases for Each Step of the Tuberculosis Care Cascade

Case Type, Cascade Step	Cases, No. (Range)	Estimation Method	Calculation			
Tuberculosis (all cases)						
Annual burden	532005 (333760–764480)	WHO 2016 time-series analysis of tuberculosis incidence in 2013 plus 50% of the number of undetected cases from 2012	Tuberculosis incidence, 2013 (all): 459000 (range, 327000–614000) [4] Tuberculosis incidence, 2012 (all): 471000 (range, 338000–627000) [4] Case detection rate, 2012: 69% (range, 52%–96%) [4] Estimated undetected cases 2012: 146000 (range, 13520–300960) 50% of undetected cases who have not died/self-cured: 73005 (range, 6760–150480)			
Accessed tests	504514 (449505–589689)	Add DS tuberculosis and RIF-R cases tested (see “DS tuberculosis, Accessed tests” and “RIF-R tuberculosis, Accessed tests” below)	DS tuberculosis cases tested: 483912 (430534–566274) RIF-R tuberculosis cases tested: 20602 (18971–23415)			
Diagnosed	435483 (411066–461766)	Add DS tuberculosis and RIF-R cases diagnosed (see “DS tuberculosis, Diagnosed” and “RIF-R tuberculosis, Diagnosed” below)	DS tuberculosis cases diagnosed: 417277 (393420–442963) RIF-R tuberculosis cases diagnosed: 18206 (17646–18803)			
Notified and treated	372577 (354162–368051)	Add DS tuberculosis and RIF-R cases notified and treated (see “DS tuberculosis, Notified and treated” and “RIF-R tuberculosis, Notified and treated” below)	DS tuberculosis cases notified and treated: 361107 (354162–368051) RIF-R tuberculosis cases notified and treated: 11470			
Successfully treated	279816 (279688–279945)	Add DS tuberculosis and RIF-R cases successfully treated (see “DS tuberculosis, Successfully treated” and “RIF-R tuberculosis, Successfully treated” below)	DS tuberculosis cases successfully treated: 274441 (274333–274549) RIF-R tuberculosis cases successfully treated: 5375 (5355–5395)			
DS Tuberculosis						
Annual burden	507533 (322078–720905)	Annual tuberculosis burden (all cases; see “Tuberculosis, Annual burden” above) less RIF-R cases	Annual tuberculosis burden: 532005 (333760–764480) RIF-R tuberculosis: 4.6% (range, 3.5%–5.7%) [2] = 24472 (11682–43575)			
Accessed tests	483912 (430534–566274)	Among bacteriologically diagnosed DS tuberculosis cases, missed cases estimated based on test type; test sensitivity to identify false-negative cases; proportion of missed cases diagnosed by culture tests and proportion treated empirically Add missed cases to total no. of DS tuberculosis cases diagnosed	Total no. of bacteriologically diagnosed DS tuberculosis cases: 289537 (272625–308278; see “DS tuberculosis cases, Diagnosed” below) No. of HIV-positive bacteriologically diagnosed cases: 178544 (168115–190101) (see “DS tuberculosis cases, Diagnosed” below) Proportion of cases tested by Xpert: 58.7% (NHLS) Proportion of cases tested by smear: 41.3% (NHLS) Xpert sensitivity: HIV-negative, 89% (81%–94%); HIV-positive, 80% (67%–88%) [12] Smear sensitivity: HIV-negative, 76% (70%–80%); HIV-positive, 50% (42%–57%) [13, 14] Proportion with culture test: HIV-positive/Xpert-negative, 14%; HIV-positive/smear-negative, 32% [16] Proportion cases treated empirically: Xpert, 21.6%; smear, 35.1% [17]; we assumed that this was 10% higher in HIV-positive cases. Estimated missed DS tuberculosis cases: 66635 (37114–123311) Calculation for midrange shown below:			
			Variable	HIV-Negative, No.	HIV-Positive, No.	Overall, No.
			Underwent bacteriological tests	110992	178544	289537
			Underwent Xpert	65192	104869	170060
			Underwent smear	45801	73676	119476
			Cases missed by Xpert	8057^aa^	26217	34275
			Cases missed by smear	14543	75164	89707
			Xpert-negative/HIV- positive with culture	…	3670	3670
			Smear-negative/HIV-positive with culture	…	24053	24053
			Underwent Xpert/received empirical treatment	…	5357	6610
			Underwent smear/received empirical treatment	…	19707	23013
			Missed cases	…	48594	66635
			^aa^Example calculation of HIV-negative cases missed by Xpert = [no. tested by Xpert/Xpert sensitivity] – [no. tested by Xpert] = [65192/0.89] – 65192 = 8057			
DS tuberculosis						
Diagnosed	417277 (393420–442963)	Pooled estimate of ILTF from published studies No. with ILTF back calculated from bacteriologically confirmed DS tuberculosis cases notified and treated No. with ILTF added to no. of DS tuberculosis cases notified and treated	No. of bacteriologically diagnosed DS tuberculosis cases notified and treated (ETR): 233367^bb^ (^bb^ETR fields were only changed to accommodate Xpert results during 2013. Using estimates from NHLS test data, the no. with positive bacteriological tests recorded in ETR was increased by 30% in pulmonary tuberculosis in patients >7 years of age, to account for underreporting of Xpert results in early 2013) ILTF estimated from pooled analysis of studies undertaken in South Africa [17–32] (see Annexure A in Supplementary Materials) Rate of ILTF for bacteriologically confirmed tuberculosis cases: 19.4% (14.4%–24.3%) [17–32] Total no. of bacteriologically diagnosed cases: 289537 (272625–308278) No. of bacteriologically diagnosed cases with ILTF: 56170 (39258–74912) (Example of midrange calculation: no. of bacteriologically confirmed cases diagnosed = 233367/80.6% = 289537; no. with ILTF = 289537 – 233367 = 56170) Add to cases notified: 361107 (354162–368051)			
Notified and treated	361107 (354162–368051)	No. of cases recorded as newly registered tuberculosis cases in ETR, adjusted for underreporting of this category	Cleaned, deduplicated data from ETR: 347218 newly registered patients with tuberculosis Adjusted to account for patients not previously newly registered and reported as moved in to district (estimated increase, 4%; range, 2%–6%)			
Successfully treated	274441 (274333–274549)	Based on mean success rate and 95% CI among adjusted no. of DS tuberculosis cases notified and treated	Cleaned, deduplicated data from the electronic tuberculosis register; used system-generated outcome Numerator: no. cured and completed treatment among newly registered and moved-in patients Denominator: no. of newly registered and moved-in patients, minus those with outcome of moved out (reported at site where they moved in) Weighted mean and 95% CI calculated at district level on the basis of definition above used by South African National Department of Health: 76.00% (range, 75.97%–76.03%)			
HIV-coinfected DS tuberculosis						
Annual burden						
HIV-positive tuberculosis	329655 (185860–509920)	WHO 2016 time-series analysis of HIV-positive tuberculosis incidence in 2013 plus 50% of the number of undetected HIV-positive tuberculosis cases from 2012	HIV-positive tuberculosis incidence, 2013: 283000 (range, 182000–406000) HIV-positive tuberculosis incidence, 2012: 301000 (range, 193000–433000) Case detection rate, 2012: 69% (range, 52%–96%) [67] Estimated HIV-positive undetected cases, 2012: 93310 (range, 7720–207840) 50% of undetected cases who have not died/self-cured: 46655 (range, 3860–103920)			
HIV-positive DS tuberculosis	314491 (179355–480855)	Annual tuberculosis burden for HIV- positive cases less RIF-R cases	Annual HIV-positive tuberculosis burden: 329655 (185860–509920) RIF-R rate assumed to be same for HIV-positive patients as for all patients: 4.6% (range, 3.5%–5.7%) [2], or 15164 (6505–29065)			
Accessed tests	305910 (273121– 354848)	Among bacteriologically diagnosed HIV-positive DS tuberculosis cases, missed cases estimated on the basis of test type, test sensitivity to identify false-negative cases, proportion of missed cases diagnosed by culture, and proportion treated empirically Add to total no. of HIV-positive DS tuberculosis cases diagnosed	Total no. of HIV-positive bacteriologically diagnosed DS tuberculosis cases: 178544 (168115–190101; from “DS tuberculosis cases, Diagnosed” above) Proportion of cases tested by Xpert: 58.7% (NHLS) Proportion of cases tested by smear: 41.3% (NHLS) Xpert sensitivity in HIV positive: 80% (67%–88%) [12] Smear sensitivity in HIV-positive: 50% (42%–57%) [13, 14] Proportion with culture performed: HIV-positive/Xpert-negative, 14%; HIV-positive/smear-negative, 32% [16] Proportion of HIV-positive cases treated empirically [17] (assumed to be 10% higher in HIV-positive cases): Xpert, 23.8%; smear, 38.6 % See “DS Tuberculosis, Accessed tests” above for calculation of HIV-positive cases with missed DS Tuberculosis: 48594 (30516–81693)			
HIV coinfected DS tuberculosis						
Diagnosed	257316 (242605– 273155)	Pooled estimate of ILTF from published studies No. with ILTF back calculated from bacteriologically confirmed HIV-positive DS Tuberculosis cases notified and treated No. with ILTF added to no. HIV-positive DS Tuberculosis cases notified and treated	No. of HIV-positive patients with bacteriologically diagnosed DS tuberculosis notified and treated (ETR): 143907 ILTF estimated from pooled analysis of studies undertaken in South Africa [17–32] (Supplementary Materials); same proportion of ILTF used for HIV-positive cases because 2 studies found no association between ILTF and HIV-negative status Rate of ILTF for bacteriologically confirmed tuberculosis cases: 19.4% (14.4%–24.3%) [17–32] Total no. of HIV-positive patients with bacteriologically diagnosed tuberculosis: 178544 (168115–190101) No. of HIV-positive patients with bacteriologically diagnosed DS tuberculosis (ETR) with ILTF: 34638 (24209–46195) (Example of midrange calculation: no. of bacteriologically confirmed cases diagnosed = 143907/80.6% = 178544; no. with ILTF = 178544–143907 = 34638) Add ILTF to 222678 (218396–226961)			
Notified and treated	222678 (218396– 226961)	Proportion of HIV-positive cases applied to adjusted no. of DS tuberculosis cases notified (see “DS tuberculosis, “Notified and Treated” above).	Cleaned, deduplicated data from the national electronic tuberculosis register (2013): HIV-positive, 192952; HIV-negative, 119949; HIV status unknown, 34317 (9.9%) Among those tested: 61.7% HIV-positive 61.7% of DS tuberculosis cases (361107; 354162–368051)			
Successfully treated	164804 (161548– 168110)	Based on mean success rate and 95% CI among adjusted no. of HIV- positive DS tuberculosis cases notified and treated	Cleaned, deduplicated data from the electronic tuberculosis register; used system-generated outcome. Numerator: no. of HIV-positive patients who were cured and completed treatment among newly registered and moved-in patients Denominator: no. of HIV-positive newly registered and moved-in patients, minus patients with outcome of moved-out (reported at site where they moved in) Weighted mean and 95% CI calculated at district level on the basis of definition above used by National Department of Health: 74.01% (range, 73.97%–74.07%)			
RIF-R tuberculosis						
Annual burden	24472 (11682– 43575)	Proportion of RIF-R cases in the annual tuberculosis burden (see “Tuberculosis, Annual burden” above)	Annual tuberculosis burden (all cases): 532005 (333760–764480) RIF-R: 4.6% (range, 3.5%–5.7%) [2]			
Accessed tests	20602 (18971– 23415)	Back calculated from RIF-R tuberculosis cases diagnosed on the basis of cases bacteriologically diagnosed, by test type and test sensitivity	RIF-R tuberculosis cases diagnosed: 18206 (17646–18803) Proportion of cases diagnosed by Xpert: 58.7% (NHLS); 10777 (10446–11131) Proportion of cases diagnosed by Genotype MDR-TBplus: 41.3% (NHLS); 7429 (7200–7673) Xpert sensitivity: *M. tuberculosis*, 88% (83%–92%); Rif-R TB, 94% (87%–97%) [12] Genotype MDR-TBplus: 98.1% (95.9%–99.1%) [15]No. of cases missed by Xpert: 2251 (1259–4284) No. of cases missed by Genotype MDR-TBplus: 144 (65–328)			
Diagnosed	18206 (17646– 18803)	Back calculated from RIF-R tuberculosis cases notified and treated and rate of ILTF	Rate of initial loss to follow-up estimated from nationally representative DR-tuberculosis survey that reported results for the 2013 cohort [34]: 37% (35–39%) (Example of midrange calculation for RIF-R tuberculosis cases diagnosed = no. receiving treatment/[100% – ILTF rate] = 11470/[100% – 37%] = 18206) ILTF: 6736 (6176–7333)			
Notified and treated	11470	Summary data from EDR	Includes no. of patients receiving MDR-TB treatment (10880) and XDR-TB treatment (590)			
Successfully treated	5375 (5355–5395)	Proportion of RIF-R tuberculosis cases notified and treated that were successfully treated	Numerator: no. of DR-tuberculosis patients who were cured and completed treatment Denominator: no. of patients with DR-tuberculosis in 2013 cohort Weighted mean and 95% CI calculated at provincial level: 46.86% (46.69%–47.04%)			

Abbreviations: CI, confidence interval; DR, drug resistant; DS, drug susceptible; EDR, electronic drug-resistant tuberculosis register; ETR, electronic tuberculosis register; HIV, human immunodeficiency virus; ILTF, initial loss to follow-up; MDR-TB, multidrug-resistant tuberculosis; *M. tuberculosis*, *Mycobacterium tuberculosis*; NHLS, South African National Health Laboratory Service; RIF-R, rifampicin resistant; WHO, World Health Organization; XDR-TB, extensively drug-resistant tuberculosis; Xpert, Xpert MTB/RIF test.

#### Burden of Tuberculosis

We used the World Health Organization 2016 time-series analysis of tuberculosis incidence to estimate the 2013 burden for all cases and for HIV-coinfected cases [4]. We defined the “tuberculosis burden” as the number of incident tuberculosis cases in 2013 plus 50% of the number of undetected cases from 2012, with the assumption that 50% of individuals with undetected tuberculosis had died or self-cured by the start of 2013. The rationale for this approach is addressed in the Discussion, below. The number of RIF-R cases was estimated from the South African tuberculosis drug resistance survey [2] and subtracted from these figures to derive figures for all DS tuberculosis cases and for HIV-coinfected DS tuberculosis cases.

#### Individuals Who Accessed Tuberculosis Tests

The gap between the number of individuals with tuberculosis who accessed tests and the number diagnosed with tuberculosis reflects cases missed by tests with a lower sensitivity than that of culture (ie, false-negative cases). The number who accessed tests was back calculated from the number who received a diagnosis, based on laboratory data on the proportion for whom tuberculosis was diagnosed by smear, Xpert, and GenoType MTBDRplus (Central Data Warehouse, National Health Laboratory Services); estimates of test sensitivity [12–15]; estimates of the proportion of smear-negative and Xpert-negative cases with a culture test [16]; and estimates of the proportion of false-negative cases that were treated empirically [17].

#### Individuals Diagnosed with Tuberculosis

The number of tuberculosis cases diagnosed was back calculated from the number of cases notified and treated, based on the rate of initial loss to follow-up (ILTF; ie, individuals with bacteriologically confirmed tuberculosis who did not initiate treatment).

We estimated the ILTF rate for DS tuberculosis cases from studies that were undertaken in South Africa and published in peer-reviewed journals during 2006–2016 (online appendix) [17–32]. We used the “metafor” R package and computed pooled estimates by using the DerSimonian-Laird method and 95% confidence intervals by using the Wald method [33]. Studies differed in the testing strategies used (smear, smear and culture, centralized performance of Xpert, and decentralized performance of Xpert), as well as in the duration after tuberculosis diagnosis when ILTF was defined. We did not expect studies to share a common effect size and therefore used data from the random-effects model. We included studies and study arms that used smear/culture or Xpert performed centrally (to reflect the status quo in 2013).

Only 2 studies reported on the association between HIV status and ILTF, and neither found a significant association [26, 32]. We therefore assumed the same rate of ILTF among HIV-coinfected DS tuberculosis cases.

For RIF-R tuberculosis cases, we used the ILTF rate reported from a large, nationally representative study for the 2013 cohort [34].

#### Individuals Notified and Treated for Tuberculosis

We used patient-level data from the ETR to estimate the number of DS tuberculosis cases (pulmonary and extrapulmonary) that were notified and for which treatment was started. Cases are recorded as being one of 3 registration types: newly registered (the patient was at this facility), moved in (the patient arrived from another facility in the district where they would have been newly registered), and transferred in (the patient arrived from another facility outside the district where they would have been newly registered). To avoid double counting, only newly registered cases would usually be included as cases notified and treated. However, we adjusted this by 4% (range, 2%–6%) to account for the net difference between patients who moved in and those who moved out at a district level that suggests that some of the patients who moved in were not previously reported as newly registered at another facility in the district.

Over 90% of patients with tuberculosis had their HIV status recorded in ETR, and among those tested, 61.7% were infected with HIV. We applied this proportion to the adjusted number of newly registered cases to derive the number of HIV-infected individuals receiving treatment for DS tuberculosis.

The number of RIF-R cases notified and treated was based on the National Department of Health’s report for the 2013 cohort (unpublished data, Research, Information, Monitoring, Evaluation, and Surveillance, TB Control and Management Cluster, National Department of Health). These included individuals with RIF-monoresistant, multidrug-resistant, pre–extensively drug-resistant, or extensively drug-resistant tuberculosis.

#### Tuberculosis Treatment Success

We used ETR line data to estimate weighted success rates (ie, the proportion of individuals who were cured or completed treatment) and 95% confidence intervals for all individuals with DS tuberculosis and for HIV-infected individuals with DS tuberculosis, at a district level. South Africa does not have a unique health identifier to assist in tracking patients who move between facilities, presenting a challenge to calculating treatment outcomes. For DS tuberculosis cases, the National Department of Health addresses this by including outcomes for all patients who were newly registered or moved in and excluding outcomes for patients who moved out (as these are reported at the facility where they moved in). We address the implications of using this method in the discussion.

For DR tuberculosis cases, we used summary provincial data to estimate weighted success rates and 95% confidence intervals, based on the National Department of Health Report for the 2013 cohort (unpublished data, Research, Information, Monitoring, Evaluation and Surveillance, TB Control and Management Cluster, National Department of Health).

**Figure 3.**
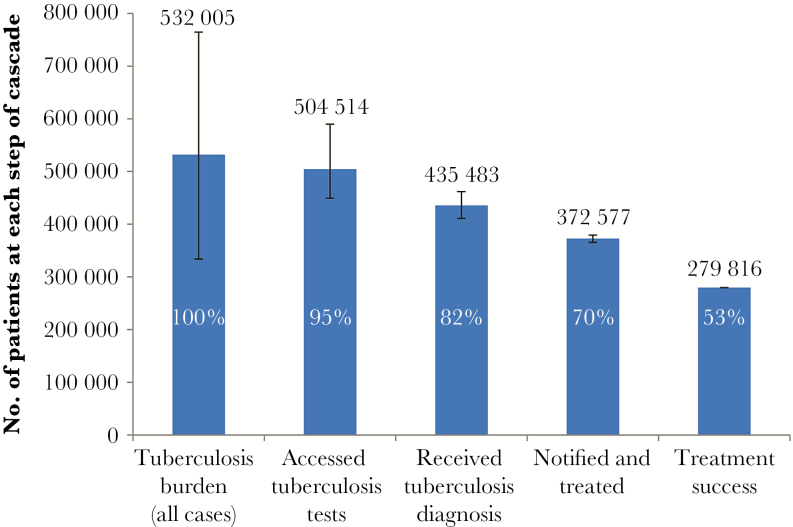
Care cascade for all patients with tuberculosis. This cascade includes patients with drug-susceptible tuberculosis and with all types of rifampicin-resistant tuberculosis. The wide confidence interval for the tuberculosis burden reflects the World Health Organisation incidence estimates for South Africa, which are based on case notification data and expert opinion on case detection gaps. The proportion at each step of the cascade is expressed in relation to the estimated burden.

**Figure 4.**
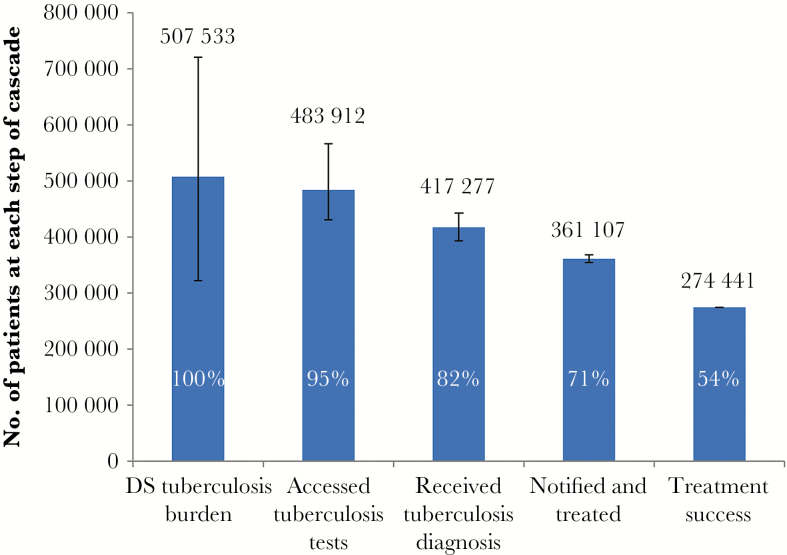
Care cascade for patients with drug-susceptible (DS) tuberculosis. The proportion at each step of the cascade is expressed in relation to the estimated burden.

**Figure 5.**
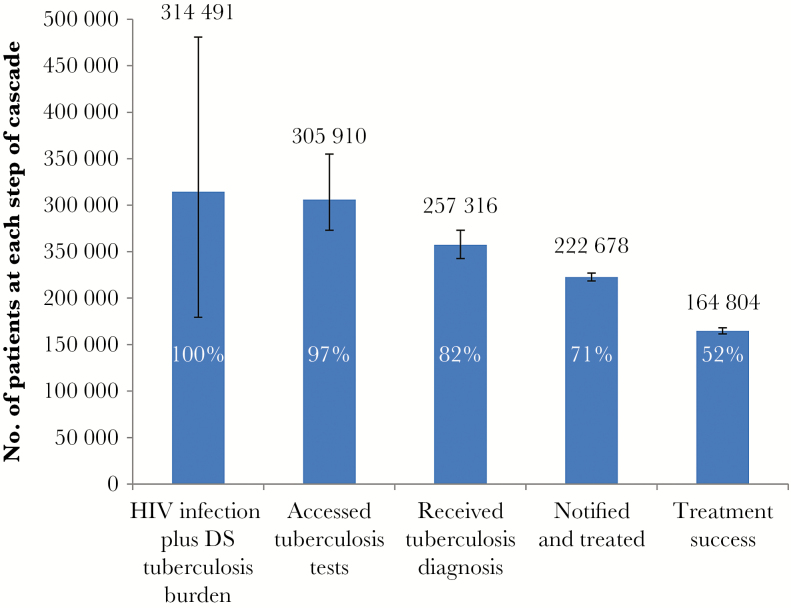
Care cascade for human immunodeficiency virus (HIV) co-infected patients with drug-susceptible (DS) tuberculosis. The proportion at each step of the cascade is expressed in relation to the estimated burden.

**Figure 6.**
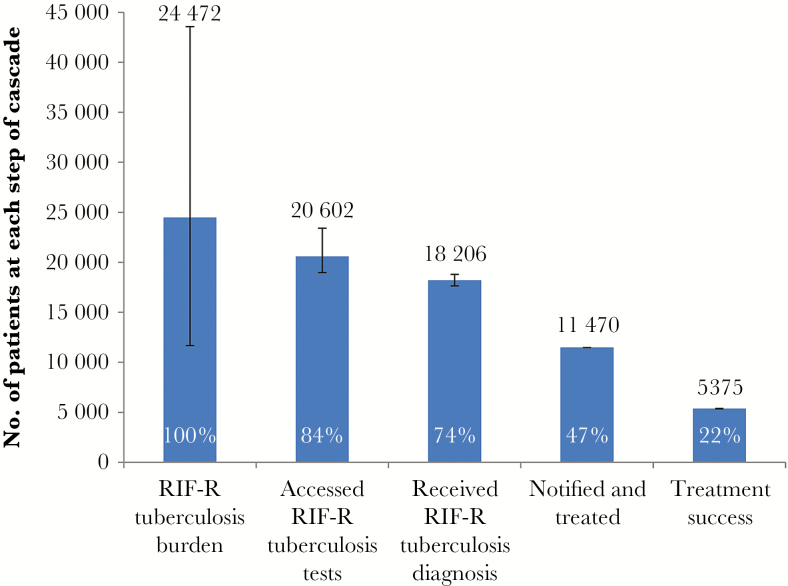
Care cascade for patients with rifampicin-resistant (RIF-R) tuberculosis. The RIF-R tuberculosis burden has a wide confidence interval, reflecting a multiplier effect when both the confidence intervals for the tuberculosis burden for all patients (derived from World Health Organization incidence estimates) and the proportion with RIF-R tuberculosis (from the national drug-resistant tuberculosis prevalence survey) are taken into account. The cascade reflects all patients with RIF-R tuberculosis (including monoresistant, multidrug-resistant, pre–extensively drug-resistant, and extensively drug-resistant tuberculosis). The proportion at each step of the cascade is expressed in relation to the estimated burden.

We express absolute numbers and the ranges of possible values and losses at each step of the cascade in relation to the estimated burden graphically in Figures 3–6.

## RESULTS

We estimated the tuberculosis burden to be 532005 cases (range, 333760–764480 cases; Figure 3). Of these, 504514 individuals (range, 449505–589689 individuals) accessed tuberculosis testing, and 435483 (range, 411066–461766) were diagnosed with tuberculosis. Among these, in 372577 (range, 354162–368051) tuberculosis was notified and treated, with 279816 (range, 279688–279945) successfully completing treatment, equivalent to 53% of the estimated tuberculosis burden. About 5% of individuals (27491) did not access diagnostic services, 13% (69030) were lost during the diagnostic process, 12% (62906) did not initiate treatment, and 17% (92761) did not successfully complete treatment.

The DS tuberculosis burden was estimated to be 507533 cases (range, 322078–720905 cases; Figure 4). Of these, 483912 individuals (range, 430534–566274 individuals) accessed tuberculosis tests, and 417277 (range, 393420–442963) were diagnosed with tuberculosis. Among these, in 361107 (range, 354162–368051) tuberculosis was notified and treated, with 274441 (range, 274333–274549) successfully completing treatment, equivalent to 54% of the estimated DS tuberculosis burden.

We estimated the burden of HIV-coinfected DS tuberculosis to be 314491 cases (range, 179355–480855 cases; Figure 5). Of these, 305910 individuals (range, 273121–354848 individuals) accessed tuberculosis tests, and 257316 (range, 242605–273155) were diagnosed with tuberculosis. Among these, in 222678 (range, 218396–226961) tuberculosis was notified and treated, with 164804 (range, 161548–168110) successfully completing treatment, equivalent to 52% of the estimated burden of HIV-coinfected DS tuberculosis burden.

The RIF-R tuberculosis burden was estimated to be 24472 cases (range, 11682–43575 cases; Figure 5). Of these, 20602 individuals (range, 18971–23415 individuals) accessed RIF-R tuberculosis tests, and 18206 (range 17646–18803) were diagnosed with RIF-R tuberculosis. Among these, in 11470 RIF-R tuberculosis was notified and treated, with 5375 (range, 5355–5395 cases) successfully completing treatment, equivalent to 22% of the estimated burden of RIF-R tuberculosis cases. About 16% of individuals with RIF-R tuberculosis (3871) did not access diagnostic services, 10% (2395) were lost during the diagnostic process, 25% (6095) did not initiate treatment, and 22% (5375) did not successfully complete treatment.

## DISCUSSION

Although the concept of continuity of care [35, 36] and attrition along the care pathway has been deliberated in tuberculosis control for many years, there are few published data that quantify outcomes [37, 38]. A study on the care cascade in India estimated that treatment was completed in 45% of prevalent tuberculosis cases in 2013 [37]. Our study estimates that just over half (53%) of all tuberculosis cases in South Africa in 2013 were successfully treated, with substantial losses during tuberculosis diagnosis, linkage to care, and retention in care.

In our setting, access to health care and tuberculosis diagnostic services does not appear to be a major impediment because testing was not accessed in only 5% of estimated cases. The small magnitude of this gap may reflect the wide network of free public primary healthcare facilities with ready access to tuberculosis diagnostic services. The National Income Dynamics Study confirmed high levels of healthcare access; in the 3 years surveyed (2008, 2010, and 2012), only 12.8% of respondents had never accessed health services, and 7% with a tuberculosis symptom (ie, cough, hemoptysis, fever, severe weight loss, and/or chest pain) in the last month had not previously accessed health services [39]. This differs from findings on the tuberculosis care cascade in India, which showed that, for more than half of the estimated 55% of cases for which treatment was not completed, diagnostic services were not accessed [37].

However, there is substantial uncertainty about the burden of disease estimate, and the magnitude of this gap in South Africa may well be underestimated, as indicated by the wide estimate range; at the upper level of the range, the gap could be as high as 34%. Estimating the annual burden of disease based on either prevalence or incidence data presents challenges. South Africa has not had a tuberculosis prevalence survey to date (the first survey is currently underway). Although tuberculosis prevalence surveys offer the best direct measure of disease burden at a point in time, estimating the annual disease burden from this would require data on the duration of disease for both HIV-infected individuals and HIV-uninfected individuals, which cannot be determined accurately. In the Indian study, the use of point prevalence data and the uncertainty in determining the 1-year period prevalence were identified as limitations [37].

Direct estimates of tuberculosis incidence are generally not financially or logistically feasible, requiring prospective cohort assessments of several hundred thousand individuals over the period of a year [40]. Indirect estimates are therefore used; for South Africa, these were based on case notification data and expert opinion on case detection gaps (ie, underdiagnosis and/or underreporting) [41].

Incidence estimates, however, fail to account for undetected cases from prior periods that were ongoing in 2013 and that contribute to the burden of cases to be identified and treated. Our method for estimating the tuberculosis burden (calculated as the number of incident cases plus 50% of the number of undetected cases from the previous year) is based on the Styblo rule that cases remain infectious for an average of 2 years, with case-fatality rates of 50% [42]. This may well be an underestimate—a systematic review of untreated pulmonary tuberculosis in HIV-uninfected individuals estimated a 3-year duration to self-cure or death [43]. On the other hand, this is balanced by HIV-coinfected individuals (62% in South Africa), who are likely to become symptomatic faster, leading to earlier detection and treatment [44].

About 13% of all cases were lost between tuberculosis testing and diagnosis, partly because of the failure to comply with diagnostic algorithms. Despite South Africa being the biggest consumer of Xpert cartridges (procuring more than half of the 7.5 million cartridges procured globally to mid-2014) [45] and the national roll-out of Xpert, <60% of individuals with presumptive tuberculosis received an Xpert test. The remainder were tested by less sensitive smear microscopy [13]. Poor compliance with the diagnostic algorithm extended to follow-on performance of culture (recommended for all HIV-infected individuals with initial negative test results). A national study undertaken during the scale-up of Xpert found that only 14% of Xpert-negative and 32% of smear-negative, HIV-infected individuals had culture performed [16]. These lapses have major implications for case detection. For example, testing 80% of presumptive tuberculosis cases with an Xpert test and 80% of Xpert-negative, HIV-infected cases with culture would reduce the number of tuberculosis cases missed at this step by almost 32000. Addressing this gap requires both routine HIV testing of presumptive tuberculosis cases and monitoring adherence to testing algorithms. Reflex laboratory testing algorithms, in which Xpert is automatically used as the first diagnostic test among presumptive tuberculosis cases and cultures are undertaken for all Xpert-negative, HIV-infected cases, could also be considered.

An estimated 12% of all cases were diagnosed but not notified and treated. These 62906 cases with ILTF exceeded the total number of tuberculosis cases notified in 2015 in 5 of South Africa’s neighboring countries combined (ie, Botswana, Namibia, Lesotho, Swaziland, and Zimbabwe). In the Indian study, the 7% with ILTF equated to 212068 cases [37]. In South Africa, the loss at this step was substantially higher among RIF-R tuberculosis cases (25%) than among DS tuberculosis cases (11%). Diagnostic delay has been reported as one of the factors contributing to ILTF [19, 22], and improved adherence to algorithms could potentially reduce delay and thus ILTF. Fragmented data systems between laboratories and health facilities contribute to poor linkage to care, and system integration can improve this. Health system failures, such as poor recording of patients’ contact details, results not being available when patients return to the health facility, and poor follow-up of patients who do not return for test results [19, 24], should be prioritized. In addition, perceptions of poor quality of services, including long waiting times and disrespectful staff attitudes, which have been well documented in the public health sector [46–51] and contribute to ILTF [52], need to be addressed. These infectious cases contribute to ongoing tuberculosis transmission and to high mortality rates. Having invested scarce financial resources in diagnosing tuberculosis, urgent efforts are required to close this gap, to avoid this fruitless expenditure.

The historical focus of the tuberculosis program on treatment success rates among new, smear-positive cases (which accounted for only one third of tuberculosis cases) has resulted in insufficient attention to outcomes for all cases. Although treatment success rates for new, smear positive cases exceed 80%, treatment success rates for all cases are substantially lower. One in every four patients initiating tuberculosis treatment did not successfully complete treatment, equivalent to the loss of 92761 individuals during treatment. It is likely that this figure is an underestimate because the method used in calculating treatment success assumes that all patients who move out of health facilities move in to facilities elsewhere. Net differences between these figures in several districts suggest that this not the case. The use of a unique patient identifier would enable improved monitoring and quantification of this loss.

Health system performance, including integration of tuberculosis and HIV services [48, 53] and strong district and facility leadership and management [54], are predictors of high treatment success rates. Poor patient knowledge, lack of empowerment among patients to engage appropriately with health providers, and high costs associated with attending daily for direct observed therapy have been found to contribute to nonadherence [48, 55–58] and need to be addressed. mHealth and other interventions to improve adherence to tuberculosis treatment need to be evaluated to reduce this significant gap.

Treatment outcomes were substantially poorer among RIF-R tuberculosis cases than among DS tuberculosis cases, with just over half the patients with RIF-R tuberculosis successfully treated. South Africa is pursuing new policy options for interventions that have been shown to improve MDR-TB treatment outcomes, including decentralized models of MDR-TB care [59, 60] and the shortened 9-month treatment regimen [61].

Although the outcome in the RIF-R tuberculosis care cascade was substantially worse than in the DS tuberculosis care cascade, the small number of cases results in minimal impact on the cascade for all tuberculosis cases. Despite the small numbers, RIF-R tuberculosis cases draw disproportionately on financial resources, with about 50% of national tuberculosis expenditures allocated to treat the 7% of patients with RIF-R tuberculosis in 2014 [62]. Unless efforts are made to reduce leakages in the RIF-R tuberculosis care cascade to help reduce the high levels of primary transmission [2, 63], this situation could be exacerbated in the future.

Overall outcomes were similar in the DS tuberculosis cascade and the HIV-coinfected DS tuberculosis cascade, suggesting parity in outcomes between individuals with and those without HIV infection. This may reflect national efforts to increase access to antiretroviral treatment.

Although the tuberculosis care cascade enumerates losses at each step, it does not reflect the delays that occur between successive steps. Two systematic reviews suggest significant time delays [64, 65]. The first of these reported overall delays of 25–185 days, with delays from symptom onset to the first healthcare visit of 5–162 days (average, 29 days) for patients and delays from the first healthcare visit to diagnosis of 2–87 days (average, 25 days) for health systems [64]. The second review reported overall delays of 21–136 days, with patient delays ranging from 7 to 69 days and health system delays ranging from 2 to 120 days [65]. The patient pathway analysis, a complementary approach that seeks to address the bottlenecks and time delays that contribute to ongoing tuberculosis transmission would add value to the current approach.

Despite relatively advanced data systems in South Africa, the absence of a unique health identifier and poor data integration makes it difficult to track individuals along the care cascade from tuberculosis testing to diagnosis, treatment initiation, and completion. We have thus estimated the number of cases at each step, and the methods we used have limitations, as already discussed. The use of a unique health identifier would enable a true cohort approach to reporting on the tuberculosis care cascade and would provide a more accurate reflection of attrition at each step.

The national findings on the tuberculosis care cascades may hide significant variations that occur at provincial, district, and facility levels. Improved data systems capable of providing this information at a more granular level would allow appropriate geographic targeting of interventions to areas where they are needed most.

In conclusion, despite the vast majority of tuberculosis cases engaging the public health system in South Africa, just over half the estimated cases were successfully treated in 2013. Health system failures at every level, from poor adherence to tuberculosis testing algorithms to poor linkage and retention in care, contribute. The analysis suggests that no single intervention will help achieve the End TB Strategy goals and that high-impact interventions are required at multiple points to improve overall outcomes.

Research and development can play a role in reducing losses, such as through an effective triage test, more-sensitive diagnostic tests, and shorter/improved treatment regimens. However, many of the factors contributing to losses in the tuberculosis care cascades reflect poor implementation of existing policies and protocols. We currently have the tools available to significantly close these gaps. The tuberculosis care cascade offers a simple, visual way to illustrate where losses occur in the care continuum and, with improved data integration, can be used to routinely track programmatic efforts to close gaps in tuberculosis diagnosis, treatment initiation, and successful treatment completion.

## Supplementary Data

Supplementary materials are available at *The Journal of Infectious Diseases* online. Consisting of data provided by the authors to benefit the reader, the posted materials are not copyedited and are the sole responsibility of the authors, so questions or comments should be addressed to the corresponding author.

## Supplementary Material

Online_supplement

Online_supplement

## Notes

***Acknowledgments*.** We thank Madhukar Pai, for guidance on the analytic approach of our study; Danielle Cazabon, for research assistance; Dewald van Deventer, for cleaning and deduplicating the ETR data set; Lindiwe Mvusi, Sicelo Dlamini, and Nevilla Somnath, for guidance on refinement of the ETR data set; David Mametje, for permission to use the ETR data; Rory Dunbar, for management of the ETR data set; Sicelo Dlamini, for access to the national Electronic Drug-Resistant Tuberculosis Register data; Ananta Nanoo and Nazir Ismail for access to National Health Laboratory Services data; and Ian Schiller and Karen R. Steingart, for technical assistance with pooled estimates of initial loss to follow-up.

***Financial support*.** This work was supported by the Bill and Melinda Gates Foundation and by the URC, through the TB Care II Project (USAID cooperative agreement AID-OAA-A-10-00021).

***Supplement sponsorship.*** This work is part of a supplement sponsored by the Bill and Melinda Gates Foundation.

***Potential conflicts of interest.*** The tuberculosis care cascade analysis was undertaken on behalf of the Bill and Melinda Gates Foundation, and the manuscript was submitted to a funded supplement of this journal. All authors have submitted the ICMJE Form for Disclosure of Potential Conflicts of Interest. Conflicts that the editors consider relevant to the content of the manuscript have been disclosed.
