# Supplementary material for: The South African Tuberculosis Care Cascade: Estimated Losses and Methodological Challenges
Source: J Infect Dis. 2017 Nov 6;216(Suppl 7):S702–13. doi: 10.1093/infdis/jix335 (PMC5853316; doi:10.1093/infdis/jix335)
Supplement: Online_supplement [file jix335_suppl_online_supplement_1.docx]

**Online supplement: A systematic review of initial loss to follow-up for drug susceptible TB cases in South Africa (2006-2016)**

A PubMed search was undertaken in April 2017 using the following terms (Tuberculosis OR TB) AND ("initial default" OR "initial loss" OR "pre-treatment loss" OR "pre-treatment default" OR “lost to follow-up” OR "treatment initiation" OR “treatment start” OR "treatment non-initiation" OR “patient registration” OR “completeness of surveillance” OR "incomplete surveillance" OR “linkage to care”).

One of the authors identified and reviewed 1032 abstracts and excluded those that were **not from South Africa**, those that did not address **initial** loss to follow-up amongst TB cases, **qualitative studies** and **reviews**, and those published **before 2006** or **after 2016**. We downloaded 31 articles for full review. Seventeen of these were excluded (see Fig) and 14 were selected for inclusion. We had previously identified 2 additional articles from searches undertaken in the Stellenbosch University online catalogue (<http://sun.worldcat.org/>) using subsets of the above terms during 2014-2016.

**Fig: Flow diagram for cases included in pooled estimates of initial loss to follow-up**

Records identified through PUBMED search
(n = 1032)

Additional manuscripts identified through through previous limited use of search terms in Stellenbosch University library catalogue
(n = 2)

Abstracts excluded
(n = 1001)

- <2006 or >2016
- Not undertaken in SA
- Reviews, perspectives (searched references for additional relevant studies. Nil identified.)
- Qualitative studies
- Patients on treatment
- Not TB

Full-text articles assessed for eligibility
(n = 31)

Full-text articles excluded
(n = 17)

- 10 - MDR-TB
- 3 - cases on treatment
- 2 - community based interventions
- 1 - Not South Africa
- 1 - Same cohort as cases in manuscript already included

Studies identified for inclusion

(n = 14)

- 12 previously identified through limited use of search terms in Stellenbosch University library catalogue

Studies included in pooled estimates
(n = 16)

Abstracts reviewed
(n = 1032)

All studies (and where applicable, study arms) were classified according to the type of diagnostic method used (smear/culture or Xpert MTBRif) and for studies using Xpert MTBRif, the location of testing (centralised or point of care) (Table). We calculated pooled estimates for initial loss to follow-up using metandi (1) for: 1) all studies/study arms; 2) studies using smear/culture; 3)studies using Xpert (centralised and point of care testing); 4)studies using point of care Xpert and 5) studies using smear/culture and centralised Xpert. The latter reflected the status quo in South Africa in 2013.

**Table: Studies used and pooled estimates of initial loss to follow-up**

| **Number in cohort** | **Number ILTF** | **Notified and initiated treatment** | **Testing method^1^** | **Notes** | **Reference** |
| --- | --- | --- | --- | --- | --- |
| 520 | 118 | 402 | 1 | 13 PHC facilities in Stellenbosch assessing introduction of a sputum register. Included cases with one or two positive smears. | (2) |
| 373 | 58 | 315 | 1 | 11 PHC facilities in Cape Town. Included smear and culture-positive cases | (3) |
| 794 | 158 | 636 | 1 | 24 PHC facilities in KwaZulu Natal Province. Included only smear-positive cases. | (4) |
| 200 | 34 | 166 | 2 | A pragmatic cluster-randomised trial embedded in the national Xpert roll-out that compared patient and programme outcomes at 40 PHC sites using either Xpert or microscopy. Used 4 week cut-off to define ILTF. | (5) |
| 174 | 26 | 148 | 1 |  |  |
| 1711 | 496 | 1215 | 1 | 122 PHC facilities in 5 provinces. Include individuals with at least one smear-positive result. Used 4 week cut-off to define ILTF. | (6) |
| 257 | 32 | 225 | 1 | A pragmatic prospective cluster-randomised trial amongst presumptive TB cases tested by Xpert or sputum microscopy/culture in one PHC facility in Khayelitsha, Cape Town. Used 3-month cut-off to define ILTF. | (7) |
| 167 | 41 | 126 | 3 |  |  |
| 267 | 101 | 166 | 1 | Culture-positive paediatric TB cases (<13 years in age) diagnosed at Tygerberg Hospital, Cape Town. Assessed proportion registered for treatment within 6-months of the positive culture result. | (8) |
| 291 | 63 | 228 | 1 | 2 PHC facilities in Cape Town. Study used capture-recapture method to compare cases with two positive smears or positive culture in laboratory results and those registered for treatment in the electronic TB register. | (9) |
| 100 | 18 | 82 | 3 | One PHC facility in Johannesburg evaluating point of care Xpert vs centralised testing undertaken in consecutive periods. Numbers are based on percentages reported (82% on treatment with point of care by 3 months vs 87% for cases tested centrally). | (10) |
| 104 | 14 | 90 | 2 |  |  |
| 721 | 250 | 471 | 1 | Quality of TB data records evaluated in 54 facilities in 3 provinces. Assessed smear-positive cases from sputum register and record of treatment initiation (in register or clinical record) | (11) |
| 185 | 15 | 170 | 3 | A pragmatic, multicentre randomised control trial at 5 PHC facilities, including 2 in South Africa. Data from all sites included. | (12) |
| 182 | 28 | 154 | 1 |  |  |
| 24 | 8 | 16 | 3 | Point of care Xpert testing for smear-negative cases at one PHC facility in Johannesburg. Cases included Xpert negative cases diagnosed on culture, second Xpert and on 3^rd^ smear. | (13) |
| 593 | 137 | 456 | 1 | Linkage to care study undertaken for TB patients diagnosed at Helen Joseph hospital in Johannesburg and referred to city clinics. | (14) |
| 66 | 7 | 59 | 1 | Three diagnostic models evaluated in 6 towns in rural Karoo, Western Cape. Study sequentially used central smear/culture, then decentralised Xpert, then centralised Xpert. | (15) |
| 77 | 1 | 76 | 3 |  |  |
| 41 | 0 | 41 | 2 |  |  |
| 72 | 9 | 63 | 3 | Point of care Xpert testing at a primary care clinic in Johannesburg with additional tests as required. Cases initiating treatment reported at 6 months. | (16) |
| 4049 | 725 | 3324 | 1 | Study undertaken in 35 high burden PHC facilities in Durban, KwaZulu Natal for cases with at least one positive sputum smear. | (17) |
|  | | | | | |
| **Testing method/s** | | | | **Pooled estimate for initial loss to follow-up (%) (95% CI)** | |
| 1,2,3 | | | | 17.1 (13.5 – 21.4) | |
| 1 | | | | 20.8 (17.0 – 25.1) | |
| 2,3 | | | | 11.5 (6.3-20.0) | |
| 3 | | | | 13.0 (6.5 – 24.1) | |
| 1,2 | | | | 18.9 (15.1 – 23.2) | |
| ^1^Testing method: 1 = smear (with or without culture), 2=centralised Xpert, 3= point of care Xpert. | | | | | |

The pooled estimates of initial loss to follow-up reported from South African studies had several limitations. The systematic search strategy used only published studies from a single data source (PubMed). We included thee studies reporting initial loss to follow-up in randomised controlled trials; these estimates may not be comparable to that found in routine practice, partly due to increased resource availability and additional efforts made to retain patients in care. Thirteen studies were undertaken under programmatic conditions and used routine data, with limitations on accuracy and completeness of records.

Several studies did not define a time-frame for non-initiation; the studies that defined this period used varying periods of between one and six months. Most studies reported only on smear or Xpert-positive and not culture-positive TB cases. Since diagnostic delay has been found to be associated with initial loss to follow-up (3,6), the exclusion of culture-positive cases from several studies may have resulted in an under-estimation of initial loss to follow-up.

**References**

1. Harbord RM, Whiting P. metandi: Meta-analysis of diagnostic accuracy using hierarchical logistic regression. Stata J. 2009;9(2):211–29.

2. Botha E, Boon S Den, Lawrence K, Reuter H, Verver S, Lombard CJ, et al. From suspect to patient : tuberculosis diagnosis and treatment initiation in health facilities in South Africa. Int J Tuberc Lung Dis. 2008; 12(8)936–941.

3. Botha E, Den Boon S, Verver S, Dunbar R, Lawrence K, Bosman M, et al. Initial default from tuberculosis treatment: how often does it happen and what are the reasons? Int J Tuberc Lung Dis. 2008;12(7):820–3.

4. Bristow CC, Dilraj A, Margot B PL. Lack of patient registration in the electronic TB register for sputum smear-positive patients in KwaZulu-Natal, South Africa. Tuberc. 2013;93(5):567–8.

5. Churchyard GJ, Stevens WS, Mametja LD, McCarthy KM, Chihota V, Nicol MP, et al. Xpert MTB/RIF versus sputum microscopy as the initial diagnostic test for tuberculosis: A cluster-randomised trial embedded in South African roll-out of Xpert MTB/RIF. Lancet Glob Heall. Open Access article distributed under the terms of CC BY-NC-ND; 2015;3(8):e450–7. Available from: http://dx.doi.org/10.1016/S2214-109X(15)00100-X

6. Claassens MM, du Toit E, Dunbar R, Lombard C, Enarson DA, Beyers N, et al. Tuberculosis patients in primary care do not start treatment. What role do health system delays play? Int J Tuberc Lung Dis. 2013;17(5):603–7.

7. Cox HS, Mbhele S, Mohess N, Whitelaw A, Muller O, Zemanay W, et al. Impact of Xpert MTB / RIF for TB Diagnosis in a Primary Care Clinic with High TB and HIV Prevalence in South Africa : A Pragmatic Randomised Trial. PLoS Med. 2014;11(11):1–12. e1001760. doi:10.1371/journal.pmed.1001760

8. du Preez, K, H. S. Schaaf, R. Dunbar, A. Swartz, K. Bissell, D. A. Enarson a. CH. Incomplete registration and reporting of culture-confirmed childhood tuberculosis diagnosed in hospital. Public Heal Action, Union [Internet]. 2011;I(1):19–24. Available from: http://www.ingentaconnect.com/content/iuatld/pha/2011/00000001/00000001/art00007

9. Dunbar R, Hest R Van, Lawrence K, Verver S, Enarson DA, Lombard C, et al. Capture-recapture to estimate completeness of tuberculosis surveillance in two communities in South Africa. Int J Tuberc Lung Dis. 2011;15:1038–43.

10. Hanrahan CF, Clouse K, Bassett J, Mutunga L, Selibas K, Stevens W, et al. The patient impact of point-of-care vs. Laboratory placement of XpertW MTB/RIF. Int J Tuberc Lung Dis. 2015;19(7):811–6.

11. Podewils LJ, Bantubani N, Bristow C, Bronner LE, Peters A, Pym A, et al. Completeness and Reliability of the Republic of South Africa National Tuberculosis (TB) Surveillance System. BMC Public Health. 2015;15(1):765. Available from: http://www.biomedcentral.com/1471-2458/15/765

12. Theron G, Zijenah L, Chanda D, Clowes P, Rachow A, Lesosky M, et al. Feasibility, accuracy, and clinical effect of point-of-care Xpert MTB/RIF testing for tuberculosis in primary-care settings in Africa: a multicentre, randomised, controlled trial. Lancet. 2013;383(9915):424–35

13. Van Rie A, Page-Shipp L, Hanrahan CF, Schnippel K, Dansey H, Bassett J, Clouse H, Scott L, Stevens W and SI. Point-of-care Xpert® MTB/RIF for smear-negative tuberculosis suspects at a primary care clinic in South Africa. Int J Tuberc Lung Dis. 2013;17(3):368–72

14. Voss De Lima Y, Evans D, Page-Shipp L, Barnard A, Sanne I, Menezes CN, et al. Linkage to Care and Treatment for TB and HIV among People Newly Diagnosed with TB or HIV-Associated TB at a Large, Inner City South African Hospital. PLoS One. 2013;8(1):1–8. e49140. doi:10.1371/journal.pone.0049140

15. Van Den Handel T, Hampton KH, Sanne I, Stevens W, Crous R, Van Rie A. The impact of Xpert((R)) MTB/RIF in sparsely populated rural settings. Int J Tuberc Lung Dis. 2015;19(4):392–8.

16. Hanrahan CF, Selibas K, Deery CB, Dansey H, Clouse K, Bassett J, et al. Time to treatment and patient outcomes among TB suspects screened by a single point-of-care xpert MTB/RIF at a primary care clinic in Johannesburg, South Africa. PLoS One 2013 Jan [cited 2013 Aug 19];8(6):e65421. Available from: http://journals.plos.org/plosone/article?id=10.1371/journal.pone.0065421

17. Cele LP, Knight S, Webb E, Tint K, Dlungwane T. High level of initial default among smear positive pulmonary tuberculosis in eThekwini health district, KwaZulu-Natal. South African J Infect Dis [Internet]. 2016;0053(May):1–3. Available from: http://www.tandfonline.com/doi/full/10.1080/23120053.2016.1128139
