# Supplementary material for: The South African Tuberculosis Care Cascade: Estimated Losses and Methodological Challenges
Source: J Infect Dis. 2017 Nov 6;216(Suppl 7):S702–13. doi: 10.1093/infdis/jix335 (PMC5853316; doi:10.1093/infdis/jix335)
Supplement: Online_supplement [file jix335_suppl_online_supplement_2.docx]

**Online supplement: A systematic review of initial loss to follow-up (ILTF) for drug susceptible TB cases in South Africa (2006-2016)**

A PubMed search was undertaken in April 2017 using the following terms (Tuberculosis OR TB) AND ("initial default" OR "initial loss" OR "pre-treatment loss" OR "pre-treatment default" OR “lost to follow-up” OR "treatment initiation" OR “treatment start” OR "treatment non-initiation" OR “patient registration” OR “completeness of surveillance” OR "incomplete surveillance" OR “linkage to care”).

One of the authors identified and reviewed 1032 abstracts and excluded those that were **not from South Africa**, those that did not address **initial** loss to follow-up amongst TB cases, **qualitative studies** and **reviews**, and those published **before 2006** or **after 2016**. We downloaded 31 articles for full review. Seventeen of these were excluded (see Fig) and 14 were selected for inclusion. We had previously identified 2 additional articles from searches undertaken in the Stellenbosch University online catalogue (<http://sun.worldcat.org/>) using subsets of the above terms during 2014-2016.

**Fig: Flow diagram for cases included in the meta-analysis of initial loss to follow-up**

Records identified through PUBMED search
(n = 1032)

Additional manuscripts identified through through previous limited use of search terms in Stellenbosch University library catalogue
(n = 2)

Abstracts excluded
(n = 1001)

- <2006 or >2016
- Not undertaken in SA
- Reviews, perspectives (searched references for additional relevant studies. Nil identified.)
- Qualitative studies
- Patients on treatment
- Not TB

Full-text articles assessed for eligibility
(n = 31)

Full-text articles excluded
(n = 17)

- 10 - MDR-TB
- 3 - cases on treatment
- 2 - community based interventions
- 1 - Not South Africa
- 1 - Same cohort as cases in manuscript already included

Studies identified for inclusion

(n = 14)

- 12 previously identified through limited use of search terms in Stellenbosch University library catalogue

Studies included in pooled estimates
(n = 16)

Abstracts reviewed
(n = 1032)

All studies (and where applicable, study arms) were classified according to the type of diagnostic method used (smear/culture or Xpert MTBRif) and for studies using Xpert MTBRif, the location of testing (centralised or point of care) (Supplementary Table 1).

**Supplementary Table 1: Studies used in the meta-analysis of initial loss to follow-up**

| **Number in cohort** | **Number ILTF** | **Notified and initiated treatment** | **Testing method^1^** | **Notes** | **Reference** |
| --- | --- | --- | --- | --- | --- |
| 200 | 34 | 166 | 2 | A pragmatic cluster-randomised trial embedded in the national Xpert roll-out that compared patient and programme outcomes at 40 PHC sites using either Xpert or microscopy. Used 4 week cut-off to define ILTF. | (17) |
| 174 | 26 | 148 | 1 |  |  |
| 520 | 118 | 402 | 1 | 13 PHC facilities in Stellenbosch assessing introduction of a sputum register. Included cases with one or two positive smears. | (18) |
| 373 | 58 | 315 | 1 | 11 PHC facilities in Cape Town. Included smear and culture-positive cases | (19) |
| 794 | 158 | 636 | 1 | 24 PHC facilities in KwaZulu Natal Province. Included only smear-positive cases. | (20) |
| 1711 | 496 | 1215 | 1 | 122 PHC facilities in 5 provinces. Include individuals with at least one smear-positive result. Used 4 week cut-off to define ILTF. | (21) |
| 257 | 32 | 225 | 1 | A pragmatic prospective cluster-randomised trial amongst presumptive TB cases tested by Xpert or sputum microscopy/culture in one PHC facility in Khayelitsha, Cape Town. Used 3-month cut-off to define ILTF. | (22) |
| 167 | 41 | 126 | 3 |  |  |
| 291 | 63 | 228 | 1 | 2 PHC facilities in Cape Town. Study used capture-recapture method to compare cases with two positive smears or positive culture in laboratory results and those registered for treatment in the electronic TB register. | (23) |
| 4049 | 725 | 3324 | 1 | Study undertaken in 35 high burden PHC facilities in Durban, KwaZulu Natal for cases with at least one positive sputum smear. | (24) |
| 66 | 7 | 59 | 1 | Three diagnostic models evaluated in 6 towns in rural Karoo, Western Cape. Study sequentially used central smear/culture, then decentralised Xpert, then centralised Xpert. | (25) |
| 77 | 1 | 76 | 3 |  |  |
| 41 | 0 | 41 | 2 |  |  |
| 100 | 18 | 82 | 3 | One PHC facility in Johannesburg evaluating point of care Xpert vs centralised testing undertaken in consecutive periods. Numbers are based on percentages reported (82% on treatment with point of care by 3 months vs 87% for cases tested centrally). | (26) |
| 104 | 14 | 90 | 2 |  |  |
| 72 | 9 | 63 | 3 | Point of care Xpert testing at a primary care clinic in Johannesburg with additional tests as required. Cases initiating treatment reported at 6 months. | (27) |
| 721 | 250 | 471 | 1 | Quality of TB data records evaluated in 54 facilities in 3 provinces. Assessed smear-positive cases from sputum register and record of treatment initiation (in register or clinical record) | (28) |
| 185 | 15 | 170 | 3 | A pragmatic, multicentre randomised control trial at 5 PHC facilities, including 2 in South Africa. Data from all sites included. | (29) |
| 182 | 28 | 154 | 1 |  |  |
| 593 | 137 | 456 | 1 | Linkage to care study undertaken for TB patients diagnosed at Helen Joseph hospital in Johannesburg and referred to city clinics. | (30) |
| 24 | 8 | 16 | 3 | Point of care Xpert testing for smear-negative cases at one PHC facility in Johannesburg. Cases included Xpert negative cases diagnosed on culture, second Xpert and on 3^rd^ smear. | (31) |
| 267 | 101 | 166 | 1 | Culture-positive paediatric TB cases (<13 years in age) diagnosed at Tygerberg Hospital, Cape Town. Assessed proportion registered for treatment within 6-months of the positive culture result. | (32) |

^1^Testing method: 1 = smear (with or without culture), 2=centralised Xpert, 3= point of care Xpert.

We used the “metafor" R package and computed pooled estimates using the DerSimonian-Laird method and 95% confidence intervals using the Wald method (33) for: 1) all studies/study arms; 2) those using smear/culture; 3) those using Xpert (centralised and point of care testing); 4) those using point of care Xpert and 5) those using smear/culture and centralised Xpert. The latter reflected the status quo in South Africa in 2013. We report estimates from both the fixed-effects and the random-effects model in Supplementary Table 2.

Studies differed in the testing strategies used (smear vs. smear and culture vs. centralised Xpert); the duration post-TB diagnosis at which ILTF was defined (which varied from 4 weeks to 6 months) and were undertaken in different contexts (intervention studies as well as routine operational conditions). We therefore did not expect studies to share a common effect size and used data from the random effects model which showed a pooled estimate for ILTF of 0.194 (95% CI 0.144 – 0.243). The I^2^ of >95% suggests considerable heterogeneity, which is to be expected based on the methodological differences described.

**Supplementary Table 2: Pooled estimates of the proportion with initial loss to follow-up with different TB testing strategies**

|  | Fixed effect model | | Random-effects model | |
| --- | --- | --- | --- | --- |
| Testing method^1^ | Pooled Proportion | 95% CI | Pooled Proportion | 95% CI |
| Testgroup (1,2,3)  N=21  I^2^ = 96.50% | 0.179 | 0.170 – 0.188 | 0.182 | 0.134 – 0.230 |
| Testgroup (1)  N=12  I^2^ = 93.63% | 0.233 | 0.222 – 0.243 | 0.216 | 0.173 – 0.260 |
| Testgroup (2,3)  N=9  I^2^ = 91.60% | 0.069 | 0.053 – 0.084 | 0.128 | 0.070 – 0.186 |
| Testgroup (3)  N=6  I^2^ = 92.34% | 0.068 | 0.050 – 0.087 | 0.146 | 0.063 – 0.228 |
| Testgroup (1,2)  N=15  I^2^ = 95.85% | 0.209 | 0.199 – 0.218 | 0.194 | 0.144 – 0.243 |
| ^1^Testing method: 1 = smear (with or without culture), 2=centralised Xpert, 3= point of care Xpert. | | | | |

The meta-analysis of initial loss to follow-up reported from South African studies has several limitations. The systematic search strategy used only published studies from a single data source (PubMed). We included three studies reporting initial loss to follow-up in randomised controlled trials; these estimates may not be comparable to that found in routine practice, partly due to increased resource availability and additional efforts made to retain patients in care. Thirteen studies were undertaken under programmatic conditions and used routine data, with limitations on accuracy and completeness of records.

Several studies did not define a time-frame for non-initiation; the studies that defined this period used varying periods of between one and six months. Most studies reported only on smear or Xpert-positive and not culture-positive TB cases. Since diagnostic delay has been found to be associated with initial loss to follow-up (19,22), the exclusion of culture-positive cases from several studies may have resulted in an under-estimation of initial loss to follow-up.
